# Supplementary material for: Microsatellite Status and IκBα Expression Levels Predict Sensitivity to Pharmaceutical Curcumin in Colorectal Cancer Cells
Source: Cancers (Basel). 2022 Feb 17;14(4):1032. doi: 10.3390/cancers14041032 (PMC8870219; doi:10.3390/cancers14041032)
Supplement: Supplementary file 1 [file cancers-14-01032-s001.zip › 00. Orginal Blots.pptx]

## Slide 1
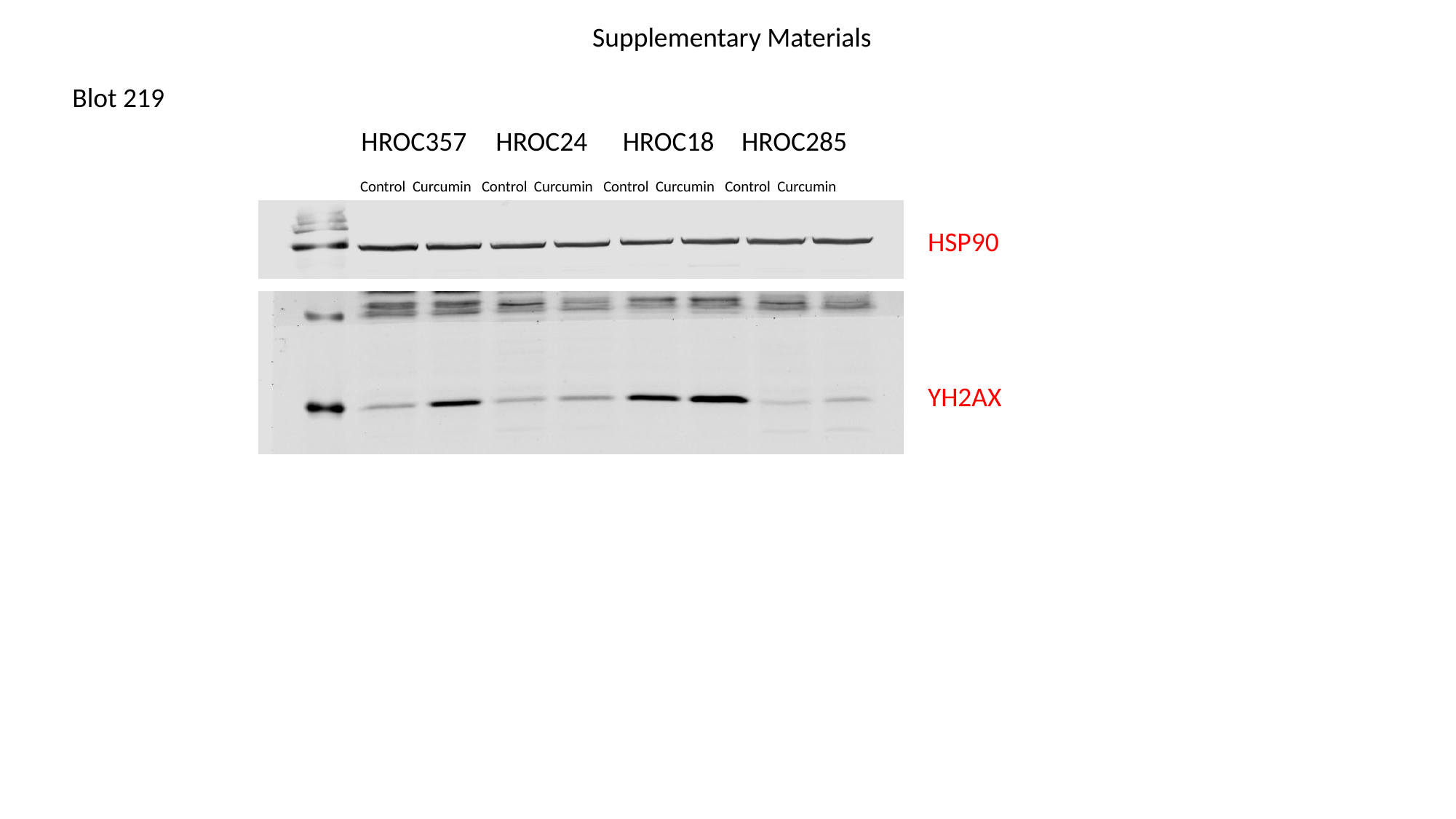

Supplementary Materials
Blot 219
HROC357
HROC24
HROC18
HROC285
Control Curcumin Control Curcumin Control Curcumin Control Curcumin
HSP90
YH2AX

## Slide 2
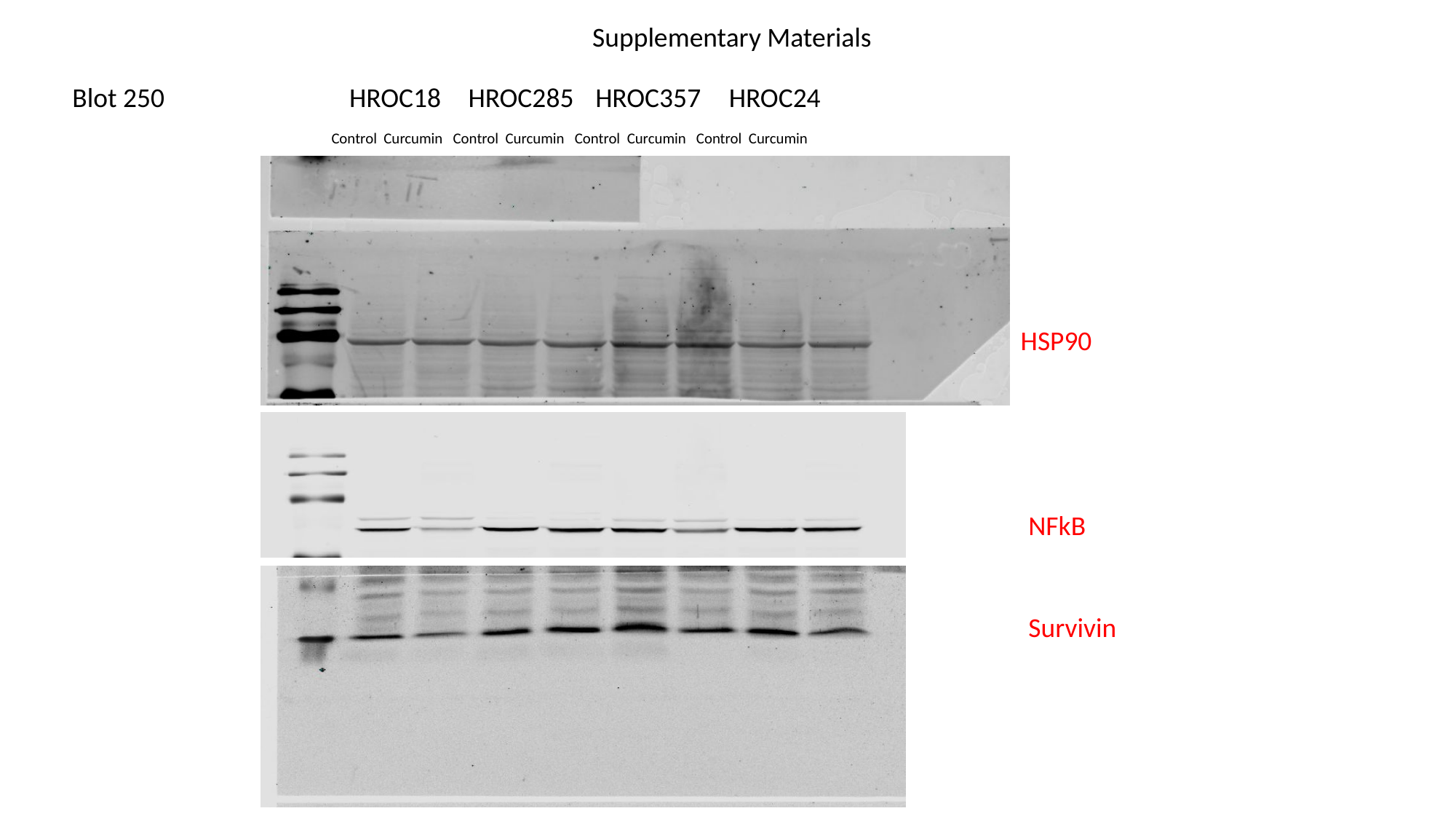

Supplementary Materials
Blot 250
HROC18
HROC285
HROC357
HROC24
Control Curcumin Control Curcumin Control Curcumin Control Curcumin
HSP90
NFkB
Survivin

## Slide 3
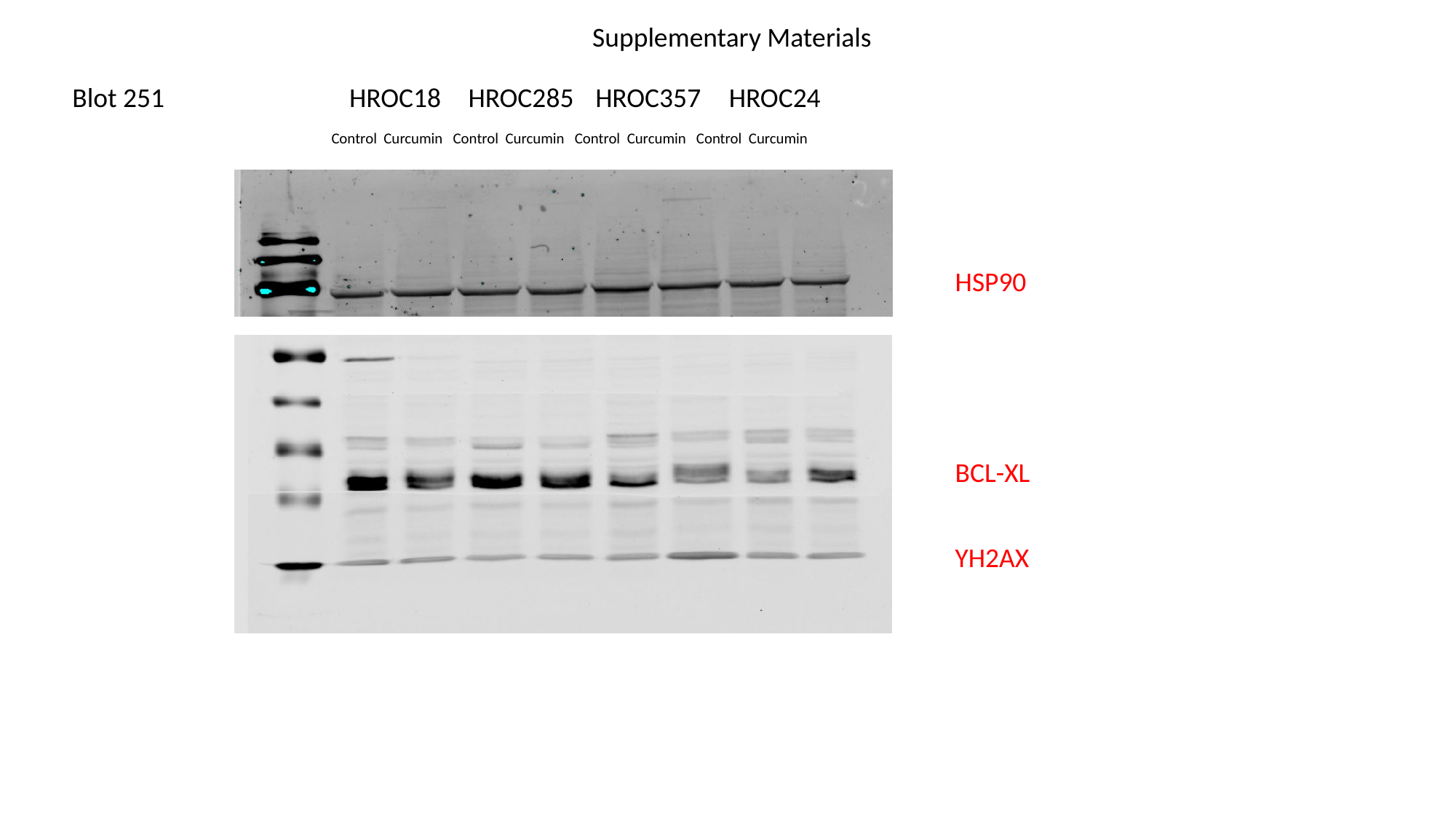

Supplementary Materials
Blot 251
HROC18
HROC285
HROC357
HROC24
Control Curcumin Control Curcumin Control Curcumin Control Curcumin
HSP90
BCL-XL
YH2AX
