# Supplementary material for: Microsatellite Status and IκBα Expression Levels Predict Sensitivity to Pharmaceutical Curcumin in Colorectal Cancer Cells
Source: Cancers (Basel). 2022 Feb 17;14(4):1032. doi: 10.3390/cancers14041032 (PMC8870219; doi:10.3390/cancers14041032)
Supplement: Supplementary file 1 [file cancers-14-01032-s001.zip › 00. Supplemenary Figures.pptx]

## Slide 1
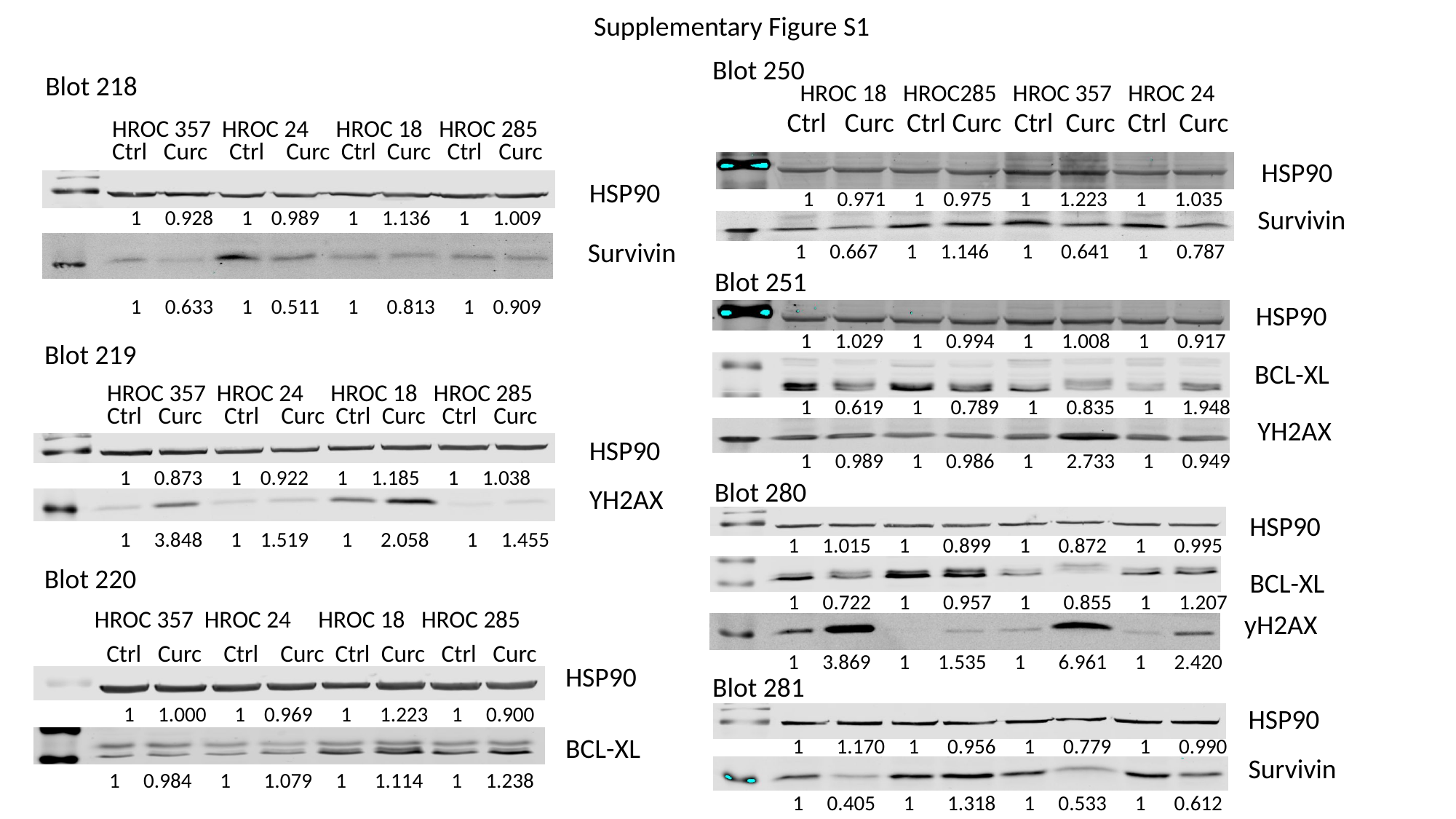

Supplementary Figure S1
Blot 250
Blot 218
HROC 18 HROC285 HROC 357 HROC 24
Ctrl Curc Ctrl Curc Ctrl Curc Ctrl Curc
HROC 357 HROC 24 HROC 18 HROC 285
Ctrl Curc Ctrl Curc Ctrl Curc Ctrl Curc
HSP90
HSP90
 1 0.971 1 0.975 1 1.223 1 1.035
Survivin
 1 0.928 1 0.989 1 1.136 1 1.009
Survivin
 1 0.667 1 1.146 1 0.641 1 0.787
Blot 251
 1 0.633 1 0.511 1 0.813 1 0.909
HSP90
 1 1.029 1 0.994 1 1.008 1 0.917
Blot 219
BCL-XL
HROC 357 HROC 24 HROC 18 HROC 285
 1 0.619 1 0.789 1 0.835 1 1.948
Ctrl Curc Ctrl Curc Ctrl Curc Ctrl Curc
YH2AX
HSP90
 1 0.989 1 0.986 1 2.733 1 0.949
 1 0.873 1 0.922 1 1.185 1 1.038
Blot 280
YH2AX
HSP90
 1 3.848 1 1.519 1 2.058 1 1.455
 1 1.015 1 0.899 1 0.872 1 0.995
Blot 220
BCL-XL
 1 0.722 1 0.957 1 0.855 1 1.207
HROC 357 HROC 24 HROC 18 HROC 285
yH2AX
Ctrl Curc Ctrl Curc Ctrl Curc Ctrl Curc
 1 3.869 1 1.535 1 6.961 1 2.420
HSP90
Blot 281
 1 1.000 1 0.969 1 1.223 1 0.900
HSP90
BCL-XL
 1 1.170 1 0.956 1 0.779 1 0.990
Survivin
 1 0.984 1 1.079 1 1.114 1 1.238
 1 0.405 1 1.318 1 0.533 1 0.612

## Slide 2
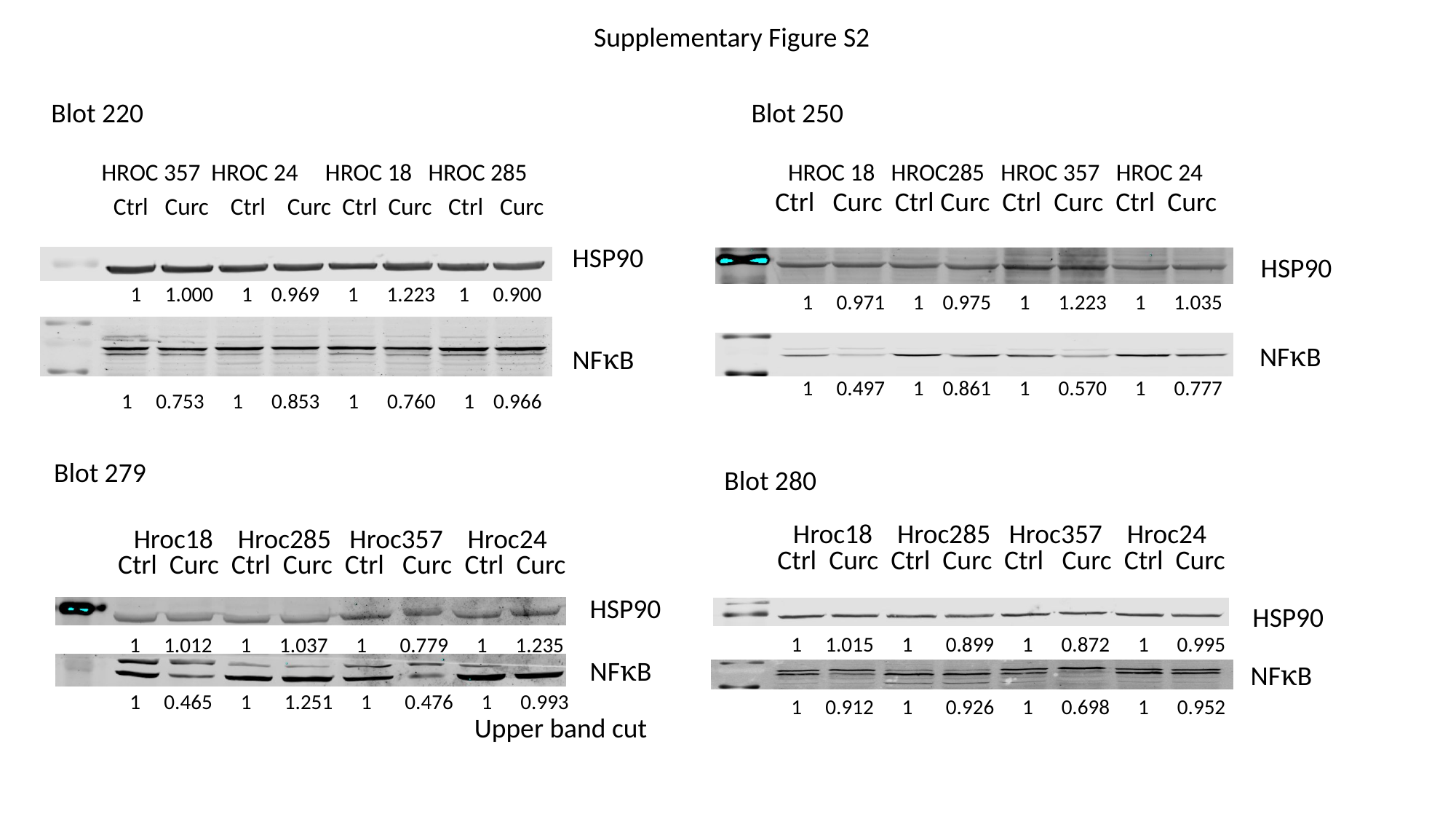

Supplementary Figure S2
Blot 220
Blot 250
HROC 357 HROC 24 HROC 18 HROC 285
HROC 18 HROC285 HROC 357 HROC 24
Ctrl Curc Ctrl Curc Ctrl Curc Ctrl Curc
Ctrl Curc Ctrl Curc Ctrl Curc Ctrl Curc
HSP90
HSP90
 1 1.000 1 0.969 1 1.223 1 0.900
 1 0.971 1 0.975 1 1.223 1 1.035
NFκB
NFκB
 1 0.497 1 0.861 1 0.570 1 0.777
 1 0.753 1 0.853 1 0.760 1 0.966
Blot 279
Blot 280
Hroc18 Hroc285 Hroc357 Hroc24
Hroc18 Hroc285 Hroc357 Hroc24
Ctrl Curc Ctrl Curc Ctrl Curc Ctrl Curc
Ctrl Curc Ctrl Curc Ctrl Curc Ctrl Curc
HSP90
HSP90
 1 1.015 1 0.899 1 0.872 1 0.995
 1 1.012 1 1.037 1 0.779 1 1.235
NFκB
NFκB
 1 0.465 1 1.251 1 0.476 1 0.993
 1 0.912 1 0.926 1 0.698 1 0.952
Upper band cut
